# Postharvest Aging and Harvest Stage Shape the Bioactive Profile, Hypolipidemic Activity, and Gut Microbiota-Modulating Effects of Citrus Peel Extracts in High-Fat-Diet-Fed Mice

**DOI:** 10.3390/foods15142470

**Published:** 2026-07-12

**Authors:** Yifan Hao, Xiaopeng Liao, Nuoying Li, Xiaoxuan Jiang, Guochuang Shan, Sisi Xie, Xingchen Li, Zhanqian Wang, Wenbin Zhang, Shejian Liang

**Affiliations:** College of Life Sciences, South China Agricultural University, Guangzhou 510642, China; 18125689686@163.com (Y.H.);

**Keywords:** citrus peel, Citri Reticulatae Pericarpium, postharvest aging, harvest stage, high-fat diet, hypolipidemic activity, gut microbiota, functional food

## Abstract

Citri Reticulatae Pericarpium (CRP), a citrus peel product derived from Citrus reticulata, is rich in flavonoids and polysaccharides, yet the effects of harvest stage and postharvest aging on its composition and metabolic functionality remain unclear. In this study, green CRP (CRP-Q) and red CRP (CRP-H) aged for 1, 3, or 5 years were extracted with 70% ethanol and evaluated in high-fat-diet (HFD)-fed mice. CRP-1Q contained the highest levels of hesperidin, nobiletin, and tangeretin, reaching 58.09 ± 0.91, 14.78 ± 0.18, and 9.15 ± 0.08 mg/g, respectively. These three flavonoids generally declined with aging, whereas longer-aged extracts showed more consistent hypolipidemic effects. CRP extracts reduced serum triglyceride and total cholesterol levels, attenuated hepatic steatosis and epididymal fat accumulation, and regulated lipid metabolism-related enzymes and genes, including FAS, ACC, HSL, LPL, SREBP-1c, HMGCR, PPAR-α, and PPAR-γ. Gut microbiota composition also changed in several aged-CRP groups, with Akkermansia abundance increasing alongside improved metabolic indicators. These findings suggest that aging duration, more than harvest stage, shaped the functional properties of CRP extracts, while the stronger activity of aged CRP was not explained by the three quantified flavonoids alone.

## 1. Introduction

Fruits and fruit-derived by-products are important sources of bioactive compounds, including flavonoids, phenolic acids, carotenoids, polysaccharides, and dietary fiber, many of which are associated with antioxidant, anti-inflammatory, and metabolic health-promoting activities [1,2]. In particular, citrus peel, a major by-product generated during fruit processing and consumption, has attracted increasing attention as a promising raw material for the development of functional foods and nutraceutical ingredients because of its abundant flavonoids and polysaccharide-like components [3].

Harvest and postharvest stages play a major role in determining the quality of bioactive extracts prepared from fruit-derived materials. The maturity stage at harvest affects phytochemical accumulation, while storage and aging after harvest may change compound stability, extractability, and transformation. As a result, the final extract may differ in chemical profile and biological activity. In citrus peel, storage or aging has been reported to modify flavonoid and polysaccharide composition, with potential effects on bioactivity [4,5,6]. These observations are of particular interest for citrus peel products such as Citri Reticulatae Pericarpium (CRP), in which prolonged aging is traditionally believed to improve quality and value. However, the relationship between aging-related compositional changes and metabolic health effects remains insufficiently understood.

With the continuous improvement in living standards, high-fat diets (HFDs) have increasingly become a common dietary pattern. Long-term consumption of HFDs, especially when combined with insufficient physical activity, can readily induce lipid metabolism disorders, obesity, and related metabolic diseases [7,8]. The liver is a central organ in maintaining lipid homeostasis through cholesterol synthesis, fatty acid biosynthesis, oxidation, and storage [9,10]. Dysregulation of these processes can lead to hyperlipidemia, a metabolic abnormality characterized by elevated serum triglycerides (TG), total cholesterol (TC), and imbalanced lipoprotein metabolism, which is an important risk factor for cardiovascular disease [11,12].

In addition to host metabolic pathways, the gut microbiota (GM) plays a critical role in nutrient digestion, energy balance, intestinal barrier maintenance, and lipid metabolism regulation [13,14]. Increasing evidence indicates that HFD-induced gut microbiota dysbiosis contributes to obesity and lipid metabolic disorders by affecting nutrient absorption, inflammatory status, and host metabolic homeostasis [15,16]. Therefore, natural food-derived ingredients capable of improving lipid metabolism while modulating gut microbiota are receiving growing attention.

Conventional lipid-lowering drugs, such as statins, are widely used in the management of hyperlipidemia, but their long-term use may be associated with adverse effects, including liver injury and gastrointestinal discomfort [17]. In this context, plant-derived bioactive ingredients are being increasingly explored as potentially safer alternatives or complementary strategies for the management of lipid metabolic disorders [18].

CRP, the dried peel of mature fruits of *Citrus reticulata* Blanco and related cultivars, is a citrus peel-derived material widely used in East Asian food and health-related practices [3]. It contains diverse bioactive compounds, particularly flavonoids and polysaccharides, which have been reported to exert lipid-lowering, anti-obesity, antioxidant, and gut microbiota-regulating activities [19,20]. Hesperidin, nobiletin, and tangeretin were used as representative flavonoid markers of citrus peel/CRP in this study. Their selection reflects their roles as major or characteristic flavonoid constituents in CRP materials from the Guangdong production region and as important marker compounds listed in the Chinese Pharmacopoeia [21]. Hesperidin is a major flavanone glycoside, whereas nobiletin and tangeretin are typical polymethoxyflavones, making these compounds suitable indicators for evaluating the flavonoid profile of CRP extracts with different harvest stages and aging durations. Consistent with their flavonoid composition, CRP extracts can reduce lipid accumulation in differentiating 3T3-L1 adipocytes and ameliorate obesity and fatty liver in HFD-fed mice [19,20]. In addition, polysaccharides from aged citrus peel have shown intestinal immunomodulatory activity, suggesting that storage duration may influence the biological properties of CRP [5]. Commercially, CRP is classified according to harvest stage into green CRP (CRP-Q) and red CRP (CRP-H), and its market value is strongly affected by aging duration. Although both harvest stage and aging are considered important quality determinants, whether they result in different hypolipidemic efficacy and gut microbiota-regulating effects remains unclear.

Therefore, this study compared CRP-Q and CRP-H extracts aged for 1, 3, and 5 years using an HFD-fed mouse model. By integrating compositional analysis, serum lipid profiling, histopathological observation, lipid metabolism-related enzyme and gene assessment, and gut microbiota analysis, we aimed to clarify how harvest stage and postharvest aging shape the metabolic functionality of CRP extracts and to evaluate their potential as value-added fruit-derived functional ingredients.

## 2. Materials and Methods

### 2.1. Chemicals

Hesperidin, nobiletin, and tangeretin reference standards with purity higher than 98% were supplied by Nanjing Bencao Yikang Biotechnology Co., Ltd. (Nanjing, China). Unless otherwise specified, analytical-grade reagents were sourced from Guangzhou Tingrui Biotechnology Co., Ltd. (Guangzhou, China). HPLC-grade methanol and acetonitrile, together with formic acid, were provided by Tianjin Yongda Chemical Reagent Co., Ltd. (Tianjin, China) Commercial assay kits for lipid metabolism-related enzymes were obtained from Quanzhou Ruixin Biological Technology Co., Ltd. (Quanzhou, China), including fatty acid synthase (FAS; cat. no. RX202423M), acetyl-CoA carboxylase (ACC; cat. no. RX202464M), hormone-sensitive lipase (HSL; cat. no. RX200099M), and lipoprotein lipase (LPL; cat. no. RX202426M). All kits were used according to the manufacturers’ instructions.

### 2.2. Preparation of CRP Extracts

Green CRP (CRP-Q) and red CRP (CRP-H) specimens representing 1-, 3-, and 5-year aging periods were prepared and stored under controlled conditions before extraction. Here, CRP materials assigned to the 1-, 3-, and 5-year groups refer to samples that underwent drying and primary processing before being aged postharvest for 1, 3, or 5 years. The 1-, 3-, and 5-year aging periods were selected to represent short-, intermediate-, and relatively long-term postharvest aging stages that are commonly encountered in CRP materials used in commercial circulation and functional food development. This design allowed comparison of compositional and biological changes across a practical aging gradient, rather than focusing on a single storage duration. The aging period was defined as the interval between completion of sample processing and subsequent extraction and analysis. The samples were ground into powder and passed through a 60-mesh sieve. The powdered CRP was extracted with 70% ethanol using ultrasound-assisted extraction at 60 °C for 120 min. The extract was filtered, concentrated under reduced pressure at 60 °C, and freeze-dried to obtain powdered CRP extracts. Sample names were assigned according to harvest stage and aging duration, resulting in the labels CRP-1Q, CRP-1H, CRP-3Q, CRP-3H, CRP-5Q, and CRP-5H. The numerical part of each label indicates the aging duration in years, whereas Q and H denote green CRP and red CRP, respectively.

### 2.3. Determination of Flavonoids and Total Sugar Content in CRP Extracts

Hesperidin, nobiletin, and tangeretin in CRP extracts were analyzed by HPLC (Agilent Technologies., Santa Clara, CA, USA). An Eclipse Plus C18 column (250 × 4.6 mm, 5 μm) was used for chromatographic separation. The mobile phase contained acetonitrile as solvent A and 0.1% formic acid in water (water/formic acid, 999/1, *v*/*v*) as solvent B. Binary gradient elution followed this program: 0–6 min, 0–30% A; 6–10 min, 30–35% A; 10–16 min, 35–60% A; 16–18 min, 60–80% A; and 18–25 min, 80–25% A, while solvent B changed correspondingly. The sample injection volume, mobile phase flow rate, and column temperature were set at 2.00 μL, 0.800 mL/min, and 40 °C, respectively. Hesperidin was monitored at 283 nm, and nobiletin and tangeretin were monitored at 340 nm. Total sugar content was determined using the phenol-sulfuric acid method and expressed as mg/g extract.

### 2.4. Animal Experimental Design

Eight-week-old male ICR mice were purchased from Guangdong Sijia Jingda Biotechnology Co., Ltd. (Guangzhou, China). All animal experiments were approved by the Experimental Animal Ethics Committee of South China Agricultural University, Guangzhou, China (approval no. 2024b007).

The mice were housed in a specific pathogen-free animal facility under controlled conditions: temperature, 24 ± 1 °C; relative humidity, 60 ± 10%; and a 12 h light/dark cycle. After acclimating for one week, the 54 mice were randomly allocated into nine groups of six animals each: control, HFD model, positive control, CRP-1Q, CRP-1H, CRP-3Q, CRP-3H, CRP-5Q, and CRP-5H.

Animals assigned to the control group were maintained on a normal diet, whereas all other groups were exposed to an HFD. Simvastatin was administered to the positive control group via oral gavage at a dose of 10 mg/kg body weight per day. CRP treatment groups received CRP extracts by oral gavage at 150 mg/kg body weight per day. This dose was selected with reference to a previous HFD-induced obese mouse study using citrus peel extract, in which oral administration at 150 mg/kg/day improved body weight gain, adipose tissue weight, serum lipid levels, and hepatic lipid accumulation [22]. The corresponding Human Equivalent Dose (HED) was calculated by body surface area conversion, using Km values of 3 for mice and 37 for adult humans. The estimated HED was 12.16 mg/kg body weight/day, corresponding to approximately 0.73, 0.85, and 1.0 g/day of CRP extract for adults weighing 60, 70, and 80 kg, respectively. A single moderate dose was used to compare CRP extracts from different harvest stages and aging durations under the same treatment condition. Equal volumes of saline were administered to both the control and model groups. The experimental intervention was carried out for 64 days, during which body weight and food intake were monitored at four-day intervals.

At the termination of the experiment, mice underwent a 12 h fasting period prior to euthanasia. Blood samples were then obtained and centrifuged at 3000 rpm for 10 min at 4 °C. The separated serum was stored at −80 °C. Fresh fecal samples were obtained under sterile conditions and preserved at −80 °C for gut microbiota profiling. Liver tissue and epididymal adipose depots were excised, weighed, rapidly snap-frozen in liquid nitrogen, and either stored at −80 °C or fixed for histological examination.

The sample size was determined according to common practice in HFD-fed mouse studies assessing lipid metabolism-related outcomes, together with the 3Rs principle of reducing animal use while maintaining biological replication. Six mice were included in each group. This group size was used in the present nine-group design to compare CRP extracts from different harvest stages and aging durations under the same intervention condition. The sample size provided biological replication for serum biochemical indices, tissue weight measurement, histological observation, lipid metabolism-related enzyme and gene analyses, and gut microbiota profiling. No animals or data points were excluded from the final analysis. Mice were monitored throughout the experiment for general health status, body weight, and food intake. Humane endpoints included severe lethargy, inability to access food or water, or marked body weight loss.

### 2.5. Serum Biochemical Analysis

Serum levels of TC, TG, LDL-C, and HDL-C were measured using a fully automated biochemical analyzer with corresponding commercial kits. Commercial assay kits were used to quantify serum FAS, ACC, HSL, and LPL levels in accordance with the manufacturers’ instructions.

### 2.6. Hematoxylin and Eosin Staining of Liver and Adipose Tissues

Liver and epididymal adipose tissues were fixed in 4% paraformaldehyde, dehydrated through a graded ethanol series, embedded in paraffin, and sectioned at 4 μm thickness. Sections were stained with hematoxylin and eosin (H&E) and observed under a light microscope. Average adipocyte area was quantified using ImageJ software version 1.53 (National Institutes of Health, Bethesda, MD, USA). Histopathological changes in liver sections were further evaluated using a semi-quantitative NAS-based scoring approach. For each treatment group, three liver sections were assessed for steatosis, lobular inflammation, and hepatocellular ballooning. Steatosis and lobular inflammation were scored from 0 to 3, while hepatocellular ballooning was scored from 0 to 2. The overall NAS was obtained by adding the scores for steatosis, lobular inflammation, and hepatocellular ballooning.

### 2.7. RT-qPCR Analysis

Total RNA was extracted from liver tissue using TRNzol Universal reagent (cat. no. DP424; Tiangen Biotech Co., Ltd., Beijing, China) according to the manufacturer’s instructions. Briefly, approximately 50 mg of liver tissue was homogenized in 1 mL of TRNzol Universal reagent. Following chloroform-induced phase separation, RNA was recovered by isopropanol precipitation, rinsed with 75% ethanol, dried briefly, and resuspended in RNase-free water.

The extracted RNA was reverse-transcribed into cDNA using the HiScript II 1st Strand cDNA Synthesis Kit with gDNA wiper (cat. no. R211-01Vazyme Biotech Co., Ltd., Nanjing, China). The reverse-transcription reaction was prepared in a final volume of 20 μL, containing 4 μL of 4 × gDNA wiper Mix, 1 μL of Oligo(dT)23VN, 1 μL of random hexamers, 1 μg of total RNA, 2 μL of 10 × RT Mix, 2 μL of HiScript II Enzyme Mix, and RNase-free water to volume. The reaction was performed at 50 °C for 15 min, followed by 85 °C for 2 min. The synthesized cDNA was stored at −80 °C until qPCR analysis.

The qPCR reaction mixture contained 5 μL of 2 × SYBR Green Pro Taq HS Premix, 0.2 μL of forward primer, 0.2 μL of reverse primer, 1 μL of cDNA, and RNase-free water to a final volume of 10 μL. The PCR program was as follows: 95 °C for 30 s, followed by 40 cycles of 95 °C for 5 s and 60 °C for 30 s. The annealing/extension temperature was 60 °C.

The hepatic mRNA levels of SREBP-1c, HMGCR, PPAR-α, and PPAR-γ were normalized to GAPDH, which was used as the internal reference gene. Primer specificity was verified using melting curve analysis. All primer pairs showed a single clear melting peak, and no obvious primer–dimer signal was observed. Primer amplification efficiencies were not determined by standard curve analysis in the original experiment. Relative transcript levels were determined using the 2^−ΔΔCt^ method. The primer sequences of the target genes and GAPDH are provided in Supplementary Table S1.

### 2.8. Gut Microbiota Analysis

Total DNA was extracted from fecal samples using a Stool Genomic DNA Extraction Kit (cat. no. D2700-50T; Beijing Solarbio Science & Technology Co., Ltd., Beijing, China) according to the manufacturer’s instructions. The amplified products were purified and sequenced on an Illumina MiSeq platform. Raw sequences were filtered using Trimmomatic, and chimeric sequences were removed using UCHIME. Operational taxonomic units (OTUs) or amplicon sequence variants were generated for downstream analysis. Alpha-diversity and beta-diversity analyses were performed using QIIME2. Taxonomic composition was analyzed at the phylum and genus levels. Spearman’s rank correlation was applied to examine the associations between dominant genera and metabolic parameters.

### 2.9. Statistical Analysis

Data are presented as the mean ± standard error of the mean (SEM). Origin 2021 was used for figure preparation. Statistical analyses were performed using SPSS version 26.0. Prior to group comparison, the distribution of each variable was assessed with the Shapiro–Wilk test, while variance homogeneity was examined using Levene’s test.

For variables meeting the assumptions of normality and equal variances, intergroup differences were analyzed by one-way ANOVA, followed by Duncan’s multiple-range test. When normality was satisfied but variances were unequal, Welch’s ANOVA was used, with the Games–Howell procedure used for multiple comparisons. For variables that did not conform to normality in one or more groups, the Kruskal–Wallis test was employed, followed by Dunn’s post hoc test.

Among all measured variables, hesperidin, nobiletin, total sugar, and PPARα were analyzed using the Kruskal–Wallis test with Dunn’s post hoc test. Tangeretin, epididymal fat weight, and HMGCR were analyzed using Welch’s ANOVA with the Games–Howell post hoc test. The remaining variables, including liver weight, final body weight, TC, TG, LDL-C, HDL-C, FAS, ACC, HSL, LPL, SREBP1c, and PPARγ, were analyzed using one-way ANOVA followed by Duncan’s multiple-range test.

For the six CRP-treated groups, harvest stage and aging duration were further entered as fixed factors in two-way ANOVA, and the harvest stage × aging duration interaction was examined in the same model. Statistical significance was accepted at *p* < 0.05.

## 3. Results

### 3.1. Comparison of Flavonoid and Total Sugar Contents Among Different CRP Extracts

The abbreviations CRP-1Q, CRP-1H, CRP-3Q, CRP-3H, CRP-5Q, and CRP-5H are used here as described in Materials and Methods, referring to green or red CRP samples after 1, 3, or 5 years of postharvest aging. The contents of hesperidin, nobiletin, tangeretin, and total sugars varied among CRP extracts from different harvest stages and aging periods (Table 1). CRP-1Q showed the highest levels of hesperidin, nobiletin, and tangeretin, with values of 58.09 ± 0.91, 14.78 ± 0.18, and 9.15 ± 0.08 mg/g, respectively. These flavonoids generally decreased with increasing aging duration. In contrast, total sugar content increased with aging, with the highest level observed in CRP-5H (427.31 ± 14.71 mg/g) and the lowest level in CRP-1Q (123.71 ± 8.93 mg/g).

At the same aging duration, CRP-Q generally contained higher levels of the measured flavonoids than CRP-H, whereas CRP-H contained higher total sugar content. These results suggest that both harvest stage and aging duration affected the measured chemical markers of CRP extracts, with aging producing an apparent shift from higher levels of the three quantified flavonoids toward higher total sugar content. Two-way ANOVA showed that the contents of hesperidin, nobiletin, tangeretin, and total sugar were significantly influenced by harvest stage and aging duration. Harvest stage × aging duration interactions were significant for the three flavonoids, but not for total sugar (Supplementary Table S2).

### 3.2. Effects of Different CRP Extracts on Body Weight and Organ Weight in Mice

**Figure 1:**
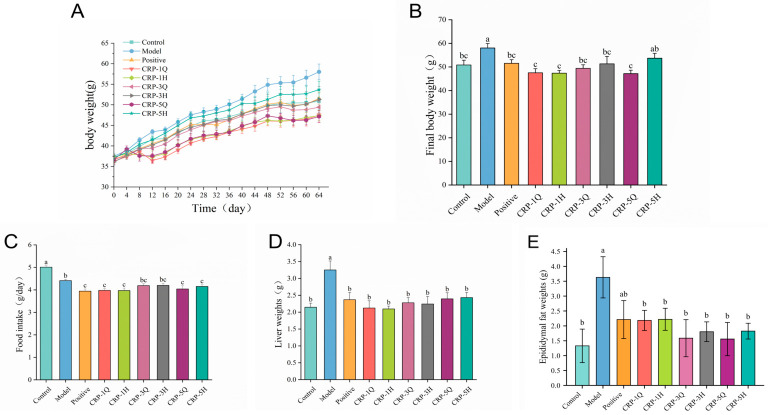
Effects of different CRP extracts on HFD-fed mice: (**A**) BW. (**B**) Final BW. (**C**) Daily average food intake. (**D**) Liver weight. (**E**) Epididymal fat weight. Different lowercase letters indicate significant differences among groups (*p* < 0.05).

During the intervention period, body weight increased more rapidly in the model group than in the control group, confirming successful establishment of the HFD-induced obesity model. CRP supplementation generally suppressed this weight gain, although the extent of the effect differed among extracts (Figure 1A). By the completion of the experiment, body weight was significantly increased in the HFD model group compared with the control group (*p* < 0.05). Relative to the HFD model group, final body weight decreased significantly after CRP-1Q, CRP-1H, and CRP-5Q treatment (*p* < 0.05). A similar downward trend was observed in the CRP-5H group, although this change did not reach statistical significance (Figure 1B).

The model group consumed less food per day than the control group, possibly due to the greater caloric density of the HFD. CRP supplementation did not increase food intake; instead, several CRP-treated groups showed slightly lower intake than the model group (Figure 1C). These results suggest that the beneficial metabolic effects of CRP extracts may not be solely explained by increased food consumption or simple dietary intake differences.

HFD feeding markedly increased liver weight and epididymal fat weight compared with the control group. Liver weight was significantly lower in mice treated with simvastatin or CRP extracts than in the HFD model group (*p* < 0.05) (Figure 1D). Similarly, all CRP extracts significantly decreased epididymal fat weight compared with the HFD model group (*p* < 0.05), with three- and five-year-aged extracts generally showing values closer to the control group than one-year-aged extracts (Figure 1E). These findings indicate that CRP extracts, particularly those subjected to longer aging, can alleviate HFD-induced hepatic enlargement and adipose tissue accumulation. Compared with the HFD model group, CRP treatment reduced final body weight by 7.46–18.73%, liver weight by 25.22–35.52%, and epididymal fat weight by 38.82–57.09%, with the larger reductions generally observed in the three- and five-year-aged CRP groups. Among the physiological parameters, epididymal fat weight was significantly influenced by aging duration in the two-way ANOVA. Liver weight, epididymal fat weight, and final body weight showed no significant harvest stage × aging duration interaction (Supplementary Table S2).

### 3.3. Effects of Different CRP Extracts on Serum Lipid Levels in Mice

**Figure 2:**
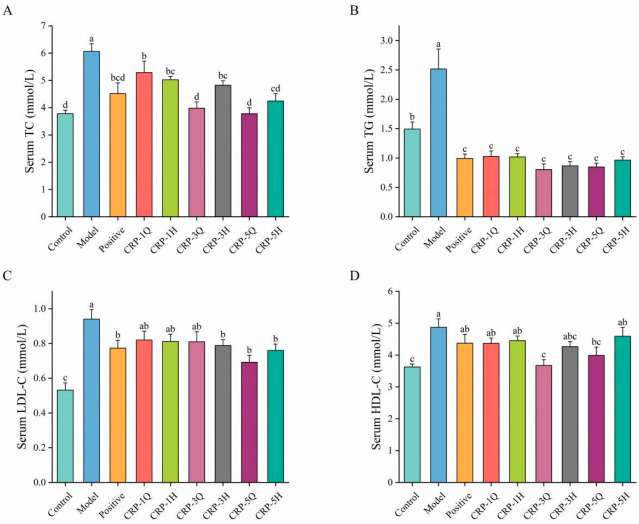
Effects of different CRP extracts on serum lipid levels in mice: (**A**) Serum TC. (**B**) Serum TG. (**C**) Serum LDL-C. (**D**) Serum HDL-C. Different lowercase letters indicate significant differences among groups (*p* < 0.05).

To evaluate the hypolipidemic potential of CRP extracts obtained from different harvest stages and aging periods, serum concentrations of TC, TG, LDL-C, and HDL-C were measured, as shown in Figure 2. Relative to the control group, the HFD model group exhibited significantly increased TC and TG levels (*p* < 0.05), confirming the development of HFD-induced dyslipidemia. Serum TC levels were significantly lower in selected CRP-treated groups than in the HFD model group (*p* < 0.05), with the extent of reduction varying among different extracts. Among the tested samples, CRP-3Q and CRP-5Q restored TC levels to values comparable with the control group, while CRP-5H also showed a marked TC-lowering effect (Figure 2A).

Serum TG levels were significantly higher in the model group than in the control group. (*p* < 0.05). All CRP extracts significantly reduced TG levels relative to the HFD model group (*p* < 0.05), and their effects were comparable to that of simvastatin (Figure 2B). Notably, TG levels in the CRP-treated groups were even lower than those in the control group, indicating a strong inhibitory effect of CRP extracts on circulating triglyceride accumulation under HFD conditions.

For LDL-C, the model group displayed the highest level among all groups. Compared with the HFD model group, LDL-C levels were significantly reduced in the positive control, CRP-1H, CRP-3H, CRP-5Q, and CRP-5H groups (*p* < 0.05), whereas CRP-1Q and CRP-3Q showed a downward trend but showed no significant difference compared with the model group based on statistical analysis (Figure 2C). Overall, compared with the HFD model group, CRP-treated groups showed reductions of 12.79–37.67% in TC, 59.21–68.01% in TG, and 12.77–26.42% in LDL-C, with more consistent changes observed in the three- and five-year-aged extracts.

HDL-C was elevated in the HFD model group compared with the control group. After CRP treatment, HDL-C levels in the CRP-3Q and CRP-5Q groups decreased toward the control level, corresponding to reductions of 24.56% and 18.10% relative to the HFD model group. This change was interpreted with caution. Rather than indicating a conventional HDL-C-lowering benefit, the shift in HDL-C appeared to reflect partial normalization of the HFD-induced lipid transport disturbance, especially when considered together with the concurrent reductions in TC, TG, LDL-C, hepatic lipid deposition, and adipose accumulation (Figure 2D). Furthermore, among serum lipid parameters, TC was significantly influenced by aging duration, whereas HDL was significantly affected by harvest stage in the two-way ANOVA. The harvest stage × aging duration interaction was not significant for TC, TG, LDL-C, or HDL-C (Supplementary Table S2).

### 3.4. Effects of Different CRP Extracts on Lipid Metabolism-Related Enzymes in Mice

**Figure 3:**
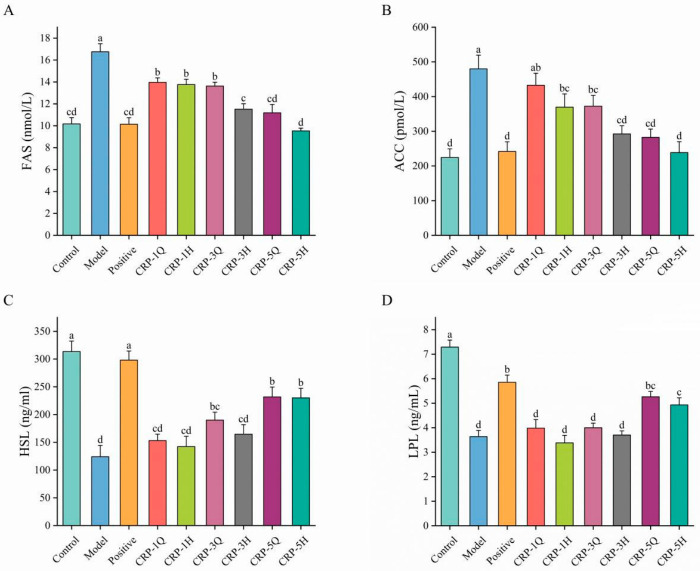
Effects of different CRP extracts on lipid metabolism-related enzymes in mice: (**A**) FAS. (**B**) ACC. (**C**) HSL. (**D**) LPL. Different lowercase letters indicate significant differences among groups (*p* < 0.05).

FAS and ACC are key enzymes involved in de novo fatty acid synthesis. As shown in Figure 3A,B, compared with the control group, HFD feeding significantly increased serum FAS and ACC levels (*p* < 0.05). Simvastatin markedly reduced both FAS and ACC levels. Serum FAS levels declined after CRP extract treatment relative to the HFD model group, with significant reductions mainly detected in the three- and five-year-aged groups (*p* < 0.05) (Figure 3A). For ACC, most CRP extracts reduced the HFD-induced elevation, although the reduction in the CRP-1Q group was not statistically significant compared with the model group (Figure 3B). These results suggest that CRP extracts can suppress fatty acid synthesis-related enzymatic alterations induced by HFD, and this effect is generally enhanced with prolonged aging.

HSL and LPL are important enzymes involved in lipid hydrolysis and triglyceride metabolism. Compared with the control group, HFD feeding significantly decreased serum HSL and LPL levels (*p* < 0.05) (Figure 3C,D). Simvastatin restored both enzymes close to control levels. Among CRP-treated groups, CRP-3Q, CRP-5Q, and CRP-5H significantly increased HSL levels compared with the model group (*p* < 0.05), whereas CRP-1Q, CRP-1H, and CRP-3H showed only limited effects (Figure 3C). For LPL, significant restoration was mainly observed in the five-year-aged CRP groups, particularly CRP-5Q and CRP-5H (*p* < 0.05) (Figure 3D). These findings indicate that longer-aged CRP extracts are more effective in promoting lipid catabolism and restoring HFD-disrupted lipid metabolism-related enzyme profiles. In terms of effect size, CRP treatment reduced FAS and ACC levels by 16.68–43.10% and 9.92–50.33%, respectively, and increased HSL and LPL levels by 14.80–87.04% and 1.82–44.77% compared with the HFD model group. Among the lipid metabolism-related enzymes, FAS, ACC, HSL, and LPL were significantly affected by aging duration in the two-way ANOVA. Harvest stage showed significant effects on FAS and ACC, with no significant harvest stage × aging duration interaction detected for these enzyme markers (Supplementary Table S2).

### 3.5. Histopathological Changes in Liver and Adipose Tissue

**Figure 4:**
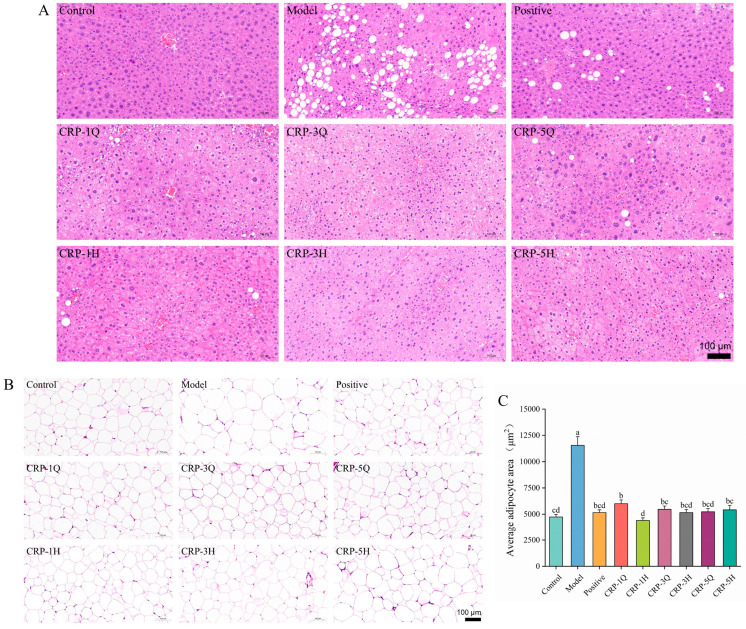
H&E staining of mouse liver and adipose tissue: (**A**) Liver (200×). (**B**) Epididymal adipose tissue (200×). (**C**) Average adipocyte area. Scale bars are indicated in the individual micrographs. Different lowercase letters indicate significant differences among groups (*p* < 0.05).

H&E staining showed pronounced hepatocellular degeneration and widespread lipid vacuolation in the liver tissues of the model group, indicating fatty liver pathology induced by the HFD. Administration of simvastatin or CRP extracts markedly improved hepatocyte morphology, as evidenced by reduced lipid droplet accumulation and smaller vacuoles (Figure 4A).

The HFD model group also exhibited significant adipocyte hypertrophy compared with the control group (*p* < 0.05). Supplementation with simvastatin or CRP extracts significantly suppressed adipocyte enlargement (*p* < 0.05), with CRP-treated groups showing 48.05–61.85% reductions in average adipocyte area compared with the HFD model group (Figure 4B,C). These results demonstrate the protective effects of CRP extracts against HFD-induced liver injury and adipocyte hypertrophy. To provide a semi-quantitative assessment of liver histopathology, steatosis, lobular inflammation, hepatocellular ballooning, and NAS total scores were further calculated from liver sections in each group (Supplementary Table S3). The HFD model group showed higher NAS total scores than the control group, mainly due to increased steatosis, lobular inflammation, and ballooning. Simvastatin and CRP treatment reduced the NAS total score to varying degrees, which was consistent with the visual improvement in hepatic lipid vacuolation shown by H&E staining. These scoring results support the histological observation that CRP extracts alleviated HFD-induced liver injury.

### 3.6. Effects of Different CRP Extracts on Lipid Metabolism-Related Gene Expression in Mice

**Figure 5:**
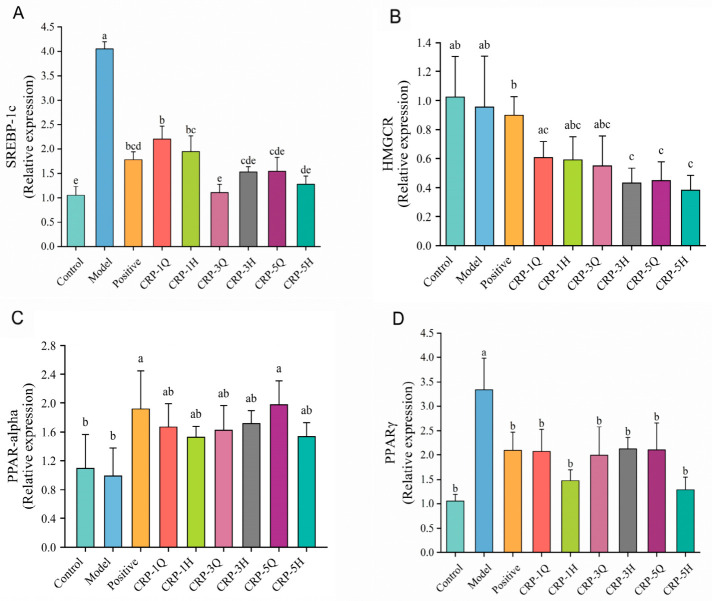
Effects of CRP extracts on lipid metabolism-related genes: (**A**) SREBP-1c. (**B**) HMGCR. (**C**) PPAR-α. (**D**) PPAR-γ. Different lowercase letters indicate significant differences among groups (*p* < 0.05).

To further explore the molecular basis of the lipid-regulating effects of CRP extracts, hepatic expression of lipid metabolism-related genes was analyzed using RT-qPCR (Figure 5). SREBP-1c, a key transcription factor involved in hepatic lipogenesis, was significantly upregulated in the model group compared with the control group (*p* < 0.05). Simvastatin and all CRP extracts reduced SREBP-1c expression to varying degrees. Among the CRP-treated groups, CRP-3Q and CRP-5H showed the strongest suppressive effects, restoring SREBP-1c expression close to the control level (Figure 5A).

HMGCR, a rate-limiting enzyme in cholesterol biosynthesis, was not significantly altered by HFD feeding compared with the control group. However, compared with the HFD model group, all CRP extracts significantly downregulated HMGCR expression (*p* < 0.05), whereas simvastatin did not show a significant reduction under the present experimental conditions (Figure 5B). These results suggest that CRP extracts may suppress hepatic cholesterol biosynthesis through HMGCR downregulation.

PPAR-α is an important regulator of fatty acid oxidation. HFD feeding did not significantly reduce PPAR-α expression compared with the control group. Nevertheless, simvastatin and CRP extracts increased PPAR-α expression, with CRP-5Q showing the highest level among all groups (Figure 5C). This indicates that CRP extracts may enhance hepatic fatty acid oxidation capacity. In contrast, PPAR-γ, which is associated with lipid storage and adipogenic processes, was significantly upregulated in the model group (*p* < 0.05). Simvastatin and all CRP extracts significantly decreased PPAR-γ expression to levels comparable with the control group (*p* < 0.05) (Figure 5D).

In comparison with the model group, CRP treatment reduced SREBP-1c, HMGCR, and PPAR-γ expression by 47.18–74.30%, 33.36–58.72%, and 33.54–62.57%, respectively, while increasing PPAR-α expression by 70.66–120.10%. Together, these findings indicate that CRP extracts regulate hepatic lipid metabolism by suppressing lipogenic and cholesterol synthesis-related pathways while enhancing fatty acid oxidation-related signaling. The two-way ANOVA showed that SREBP1c, HMGCR, and PPARα responded significantly to both aging duration and harvest stage. Significant harvest stage × aging duration interactions were detected for these genes, whereas PPARγ showed no significant response to either factor or their interaction (Supplementary Table S2).

### 3.7. Effects of Different CRP Extracts on Gut Microbiota in Mice

**Figure 6:**
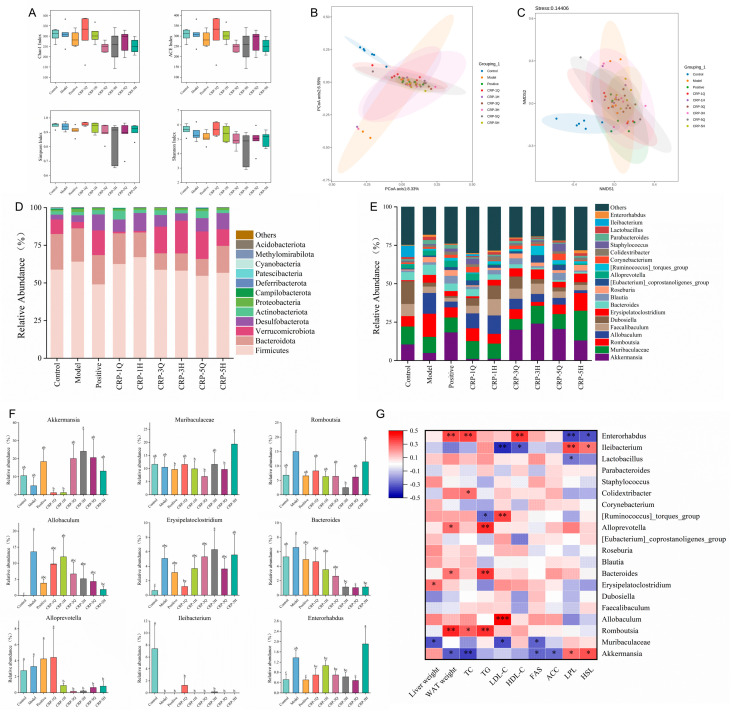
Effects of different CRP extracts on GM in mice: (**A**) α−diversity indices. (**B**) PCoA analysis (β−diversity). (**C**) NMDS analysis (β−diversity). (**D**) Relative abundance at the phylum level. (**E**) Relative abundance at the genus level. (**F**) Selected genus-level relative abundance (Different lowercase letters indicate significant differences among groups). (**G**) Spearman’s correlation analysis of the top 20 genera with hyperlipidemia indicators (* *p* < 0.05, ** *p* < 0.01, *** *p* < 0.001.).

To examine whether gut microbiota changes accompanied the lipid-lowering effects of CRP extracts, fecal samples were analyzed by 16S rRNA sequencing. Alpha-diversity indices, including Chao1, ACE, Simpson, and Shannon indices, showed no marked differences among groups (Figure 6A), indicating that CRP supplementation did not substantially alter overall microbial richness or diversity under the present conditions.

Principal coordinate analysis (PCoA) and non-metric multidimensional scaling (NMDS) based on beta-diversity showed that the gut microbial structure of the control group differed from that of the HFD model group, suggesting that HFD feeding altered gut microbiota composition. CRP-treated groups showed partial shifts in microbial community structure, although clear separation among all CRP groups was not observed (Figure 6B,C). These results indicate that CRP treatment was associated mainly with changes in selected microbial taxa, rather than with broad alterations in overall microbial diversity.

Firmicutes and Bacteroidota were identified as the most abundant bacterial phyla at the phylum level in all groups, with Verrucomicrobiota and other low-abundance phyla following thereafter (Figure 6D). Compared with the model group, several aged-CRP groups showed an increased proportion of Verrucomicrobiota, which was largely associated with changes in the genus Akkermansia. At the genus level, HFD feeding altered the relative abundance of several dominant taxa, including Akkermansia, Romboutsia, Allobaculum, Bacteroides, and Alloprevotella (Figure 6E,F).

Notably, Akkermansia abundance was increased in the positive control and in several aged-CRP groups, particularly CRP-3Q, CRP-3H, and CRP-5Q. In contrast, genera such as Bacteroides and Alloprevotella tended to decrease in three- and five-year-aged CRP groups. Romboutsia was relatively abundant in the model group and was reduced in several CRP-treated groups, although the response varied among extracts (Figure 6F).

Spearman’s correlation analysis was used to evaluate the relationships between dominant genera and metabolic indicators (Figure 6G). Akkermansia was negatively correlated with several hyperlipidemia-related parameters and positively correlated with lipid catabolism-related enzyme activities, including LPL and HSL. Conversely, genera such as Romboutsia, Allobaculum, Bacteroides, and Alloprevotella showed positive correlations with selected lipid disorder-related indicators. These results suggest that the metabolic improvements observed after CRP treatment may be partly associated with shifts in selected bacterial taxa, especially the enrichment of Akkermansia and the reduction in certain HFD-associated genera. However, these association-based findings do not establish a causal role of gut microbiota in the lipid-lowering effects of CRP extracts. The two-way ANOVA identified significant aging duration effects on Chao1, ACE, Shannon, Akkermansia, and Bacteroides among the gut microbiota-related indicators. No significant effects of harvest stage or harvest stage × aging duration interaction were detected for the selected α-diversity indices or bacterial genera (Supplementary Table S2).

## 4. Discussion

Fruit processing by-products are gaining attention as valuable sources of bioactive compounds for functional food applications. Citrus peel is particularly attractive in this context, as it contains flavonoids, polymethoxyflavones, polysaccharides, volatile constituents, and other secondary metabolites that have been linked to antioxidant, anti-inflammatory, and metabolism-supporting effects [23]. In the present study, CRP was considered not only as a traditional medicinal material but also as a fruit-derived functional ingredient. Its bioactive composition and metabolic effects were examined across different harvest stages and postharvest aging durations.

The compositional analysis showed that CRP-Q extracts generally contained higher levels of hesperidin, nobiletin, and tangeretin than CRP-H extracts at the same aging duration. In contrast, CRP-H extracts showed higher total sugar content. During aging, the measured flavonoids decreased, while total sugar content increased. Previous studies have reported that storage years can reshape the chemical profile of citrus peel, including flavonoids, phenolic acids, polysaccharide-related components, volatile constituents, fatty acids, and other secondary metabolites [24,25,26,27,28]. LC-MS- and UPLC-QTOF-MS-based metabolomic studies have also shown that Chenpi samples with different storage years can be distinguished by their global metabolite profiles, although the changing patterns of individual compounds may differ across citrus varieties, storage durations, processing conditions, and analytical methods [25,26,27]. Aging should therefore be viewed as a postharvest transformation process that remodels the whole phytochemical matrix rather than a process affecting one or two marker compounds alone.

No stable correspondence was found between the lipid-lowering effects of CRP extracts and the measured levels of hesperidin, nobiletin, or tangeretin. These compounds were selected as representative and pharmacopoeia-related markers of CRP, rather than as constituents already proven to drive the metabolic response. Total sugar content also gives only a rough compositional signal. It does not distinguish polysaccharide fractions with different molecular weights, monosaccharide profiles, glycosidic linkages, conformations, or biological activities. A cause-and-effect relationship cannot be established solely from the quantified flavonoids and the observed biological activity. For this reason, the biological differences among CRP extracts should not be assigned to hesperidin, nobiletin, tangeretin, or total sugars alone.

The stronger effects observed in the longer-aged CRP groups appeared while hesperidin, nobiletin, and tangeretin declined with aging. This inverse pattern weakens a simple explanation centered on these three flavonoids. Other parts of the CRP phytochemical matrix may have contributed, including minor flavonoids, phenolic acids, polymethoxyflavone derivatives, essential-oil or volatile constituents, terpenes, soluble sugar-related fractions, polysaccharide fractions, and aging-derived transformation products. Some compounds may accumulate during storage, whereas others may degrade, oxidize, demethylate, hydrolyze, polymerize, or form new derivatives. Their roles were not resolved in this work. A more complete chemical explanation will require expanded HPLC fingerprinting, targeted and untargeted LC-MS/MS or UPLC-QTOF metabolomic profiling, GC-MS analysis of volatile constituents, polysaccharide structural characterization, and activity-guided validation of defined fractions or individual compounds.

Among these uncharacterized aging-related changes, soluble sugar- or polysaccharide-related fractions may be particularly relevant. The increase in total sugar content suggests that soluble sugar- or polysaccharide-related fractions changed during storage. These fractions may influence lipid metabolism through effects on intestinal viscosity, bile acid binding, gut microbial fermentation, or short-chain fatty acid production. However, total sugar content is only a bulk index and cannot identify the active polysaccharide fraction. Phenolic compounds may also be transformed during aging. Oxidation, hydrolysis, demethylation, or polymerization may generate phenolic or flavonoid-derived products with biological activities different from those of the parent compounds. In addition, the activity of aged CRP extracts may reflect combined effects among multiple constituents rather than the action of one dominant compound. Interactions among flavonoids, polysaccharide-related fractions, phenolic transformation products, volatile constituents, and microbial metabolites may jointly affect lipid absorption, hepatic lipid synthesis, lipid oxidation, and gut microbial composition. These possibilities remain hypothetical in the present study and require metabolomic profiling, polysaccharide structural analysis, and activity-guided validation.

HFD feeding induced typical metabolic disturbances, including increased body weight, hepatic enlargement, epididymal fat accumulation, elevated serum TC and TG levels, and severe hepatic lipid deposition. These results are consistent with previous reports showing that prolonged HFD consumption readily disrupts lipid metabolic homeostasis and promotes obesity, hyperlipidemia, and fatty liver [7,8,29]. CRP extracts alleviated these changes to varying degrees. Serum TG levels were reduced in all CRP-treated groups, while TC levels improved more clearly in the three- and five-year-aged groups. The HDL-C response requires careful interpretation. In the present study, HDL-C was elevated in the HFD model group, whereas CRP-3Q and CRP-5Q reduced HDL-C toward the level observed in the control group. This result should not be interpreted as evidence that lowering HDL-C is inherently favorable. Under the HFD-induced dyslipidemic condition used here, the decrease in HDL-C was considered part of a broader movement toward lipid metabolic normalization, together with lower TC, TG, and LDL-C levels and improved tissue lipid deposition. Recent studies have also emphasized that HDL-C concentration alone does not fully represent HDL function or cardiometabolic protection. HDL particle composition, cholesterol efflux capacity, and anti-inflammatory or antioxidant function may provide more meaningful information than HDL-C level alone [30]. Therefore, the HDL-C changes observed in this study should be interpreted as a context-dependent response rather than as an independent beneficial endpoint.

Lipid metabolism-related enzymes provided further mechanistic information. FAS and ACC are key enzymes involved in fatty acid biosynthesis. Their upregulation can accelerate lipid accumulation in liver and adipose tissues [31,32]. HFD markedly increased FAS and ACC levels, whereas CRP extracts, especially the longer-aged extracts, suppressed these increases. HSL and LPL participate in triglyceride hydrolysis and lipid utilization. HSL hydrolyzes stored triglycerides and releases free fatty acids for energy supply. LPL facilitates the hydrolysis of circulating triglycerides and promotes fatty acid uptake and oxidation [33,34,35]. The HFD-induced decreases in HSL and LPL were partially reversed by CRP extracts, with five-year-aged CRP extracts showing the strongest effects on LPL restoration. These enzyme-level changes suggest that aged CRP extracts may reduce lipid accumulation by suppressing fatty acid synthesis and promoting lipid catabolism. They should still be interpreted as downstream biological responses to whole CRP extracts, rather than direct evidence that a specific compound was responsible for the effect.

Histopathological observations further supported the biochemical findings. HFD caused pronounced hepatic steatosis and adipocyte hypertrophy, both of which were ameliorated after CRP treatment. Excessive fat accumulation in liver and adipose tissue is a central feature of HFD-induced obesity and related metabolic disorders [29,36]. The improvements in tissue morphology in the CRP-treated groups were consistent with the reductions in serum lipids, lipogenic enzymes, and tissue weights.

At the gene expression level, CRP extracts suppressed SREBP-1c and PPAR-γ expression and increased PPAR-α expression. SREBP-1c is a key transcription factor that drives hepatic lipogenesis through the upregulation of downstream enzymes involved in fatty acid synthesis, including FAS and ACC [32,37,38,39]. Its suppression by CRP extracts was consistent with the lower lipogenic enzyme levels and reduced hepatic lipid accumulation. PPAR-γ is closely associated with adipogenesis and lipid storage, whereas PPAR-α promotes fatty acid oxidation by enhancing the transcription of genes related to β-oxidation [40,41,42]. The downregulation of PPAR-γ and upregulation of PPAR-α together indicate a shift from lipid storage toward lipid oxidation. In addition, all CRP extracts downregulated HMGCR expression, suggesting potential suppression of hepatic cholesterol biosynthesis. Since HFD did not significantly upregulate HMGCR in this study, the CRP-induced reduction should be considered an additional cholesterol synthesis-suppressing effect rather than a reversal of HFD-induced HMGCR activation. HMGCR is the rate-limiting enzyme in cholesterol biosynthesis and plays a central role in hepatic cholesterol homeostasis [43].

The present gene panel covered several central regulators of lipid metabolism, but it did not include all nodes of the proposed pathway. AMPK, for example, is an upstream energy-sensing kinase that can influence lipid synthesis and fatty acid oxidation through targets such as ACC and SREBP-1c. The reduction in ACC and SREBP-1c observed here is compatible with this regulatory direction, although AMPK activation was not measured and cannot be claimed. ACOX1 is another relevant gene, as it participates in peroxisomal β-oxidation and is often linked to PPAR-α-regulated fatty acid catabolism. The increased PPAR-α expression in CRP-treated groups suggests a shift toward lipid oxidation, but ACOX1 expression would be needed to verify whether the downstream β-oxidation program was activated. Similarly, FASN, the gene encoding FAS, was not quantified at the mRNA level, although FAS enzyme levels were reduced after CRP treatment. C/EBPα also deserves attention because it cooperates with PPAR-γ during adipocyte differentiation and lipid storage. The downregulation of PPAR-γ and the improvement of adipocyte hypertrophy suggest a possible weakening of the adipogenic program, but this interpretation remains incomplete without C/EBPα and other adipogenesis-related markers. Thus, the current results support a regulatory pattern involving lower lipogenesis, enhanced lipid catabolism, and reduced adipose lipid storage, while the AMPK–ACC/SREBP-1c/FASN, PPAR-α–ACOX1, and PPAR-γ–C/EBPα axes should be examined in future work.

Citrus peel contains several polymethoxyflavones, among which nobiletin and tangeretin have received particular attention in lipid metabolism research. Reported effects of nobiletin and related polymethoxyflavones include regulation of hepatic lipid metabolism, adipose-tissue energy expenditure, cholesterol and bile acid metabolism, and gut microbial composition [44,45,46]. These reported activities are directionally consistent with the lower levels of FAS, ACC, SREBP-1c, and PPAR-γ and the higher expression of PPAR-α observed in the CRP-treated groups. Even so, the present data do not prove a direct contribution from nobiletin, tangeretin, or any single polymethoxyflavone. The activity of whole CRP extracts is more likely to reflect the combined effects of multiple chemical fractions.

The concentrations of parent compounds in CRP extracts may not fully represent biological exposure to citrus flavonoids after ingestion. Intestinal hydrolysis, phase II conjugation, and microbial biotransformation can generate metabolites whose physicochemical and biological properties differ from those of the original flavonoids [45,47]. Polymethoxyflavones may also undergo demethylation, producing derivatives that retain or modify the activities of their parent compounds [45]. Thus, the declining concentrations of nobiletin and tangeretin during aging do not necessarily exclude the involvement of flavonoid-derived metabolites in the observed responses. Circulating metabolites and microbial transformation products were not measured in this study, leaving the proposed relationship between flavonoid metabolism and lipid metabolic responses unresolved.

Gut microbiota is strongly associated with host lipid metabolism, energy balance, and metabolic inflammation [41,48]. In this study, CRP extracts did not markedly alter alpha-diversity indices, but changes were observed in the abundance of selected taxa. This pattern is not unexpected, as dietary interventions may influence microbial community composition without necessarily producing large shifts in overall richness or diversity [49,50]. In several longer-aged CRP extract groups, Akkermansia abundance was higher than that in the HFD model group. Previous studies have associated Akkermansia with intestinal barrier function, inflammatory status, metabolic health, and obesity-related risk [51,52,53]. In the present correlation analysis, Akkermansia was negatively associated with several hyperlipidemia-related indicators and positively associated with lipid catabolism-related enzymes. In contrast, genera such as Romboutsia, Bacteroides, and Alloprevotella were positively correlated with several metabolic disorder-related parameters. Previous studies have linked Romboutsia to hyperlipidemia- and diabetes-associated microbial profiles, while increased abundance of some HFD-associated genera may reflect gut ecological imbalance under metabolic stress [52,54]. Taken together, these findings show that the lipid-lowering effects of CRP extracts were accompanied by changes in selected gut microbial taxa, including Akkermansia. Since the microbiota results were derived from 16S rRNA sequencing and correlation analysis, they should therefore be regarded as associative rather than as direct evidence supporting a microbiota-mediated mechanism.

More consistent improvements in metabolic outcomes were observed with the three- and five-year-aged CRP extracts than with their shorter-aged counterparts. The influence of harvest stage was comparatively limited. Differences between CRP-Q and CRP-H were evident in the concentrations of hesperidin, nobiletin, and tangeretin and in total sugar content. Extracts sharing the same aging duration nevertheless showed broadly comparable hypolipidemic effects. With longer aging, improvements became more consistent across serum lipid profiles, lipid metabolism-related enzymes and genes, tissue lipid deposition, and the relative abundance of selected gut microbial taxa. Within the present experimental design, this overall pattern supports enhanced metabolic functionality of CRP extracts after postharvest aging, while the contribution of gut microbiota changes remains to be verified by further mechanistic studies.

This enhancement occurred alongside progressive decreases in hesperidin, nobiletin, and tangeretin, rather than changes in the same direction as biological activity. The stronger effects of the longer-aged extracts cannot be adequately explained by these three quantified flavonoids. Other chemical changes may contribute, including increases in unquantified secondary metabolites, formation of aging-derived transformation products, structural remodeling of polysaccharide fractions, and interactions among multiple chemical groups [25,26,27,28]. The exact chemical basis remains to be clarified.

Several limitations should be noted. First, chemical characterization in this study covered only hesperidin, nobiletin, tangeretin, and total sugar content. A substantial part of the complex CRP phytochemical composition was not resolved. Other flavonoids, phenolic acids, individual polysaccharide fractions, volatile or essential-oil constituents, and aging-derived transformation products were not systematically characterized. Total sugar content, as a bulk compositional measure, provides little information on the structural and functional properties of individual polysaccharides. The metabolic differences observed among CRP extracts should not be causally assigned to the three quantified flavonoids or to total sugars alone. Although stronger metabolic functionality was observed after postharvest aging, the available chemical data do not identify the constituents or chemical fractions associated with this change. Future studies should combine expanded HPLC fingerprinting, targeted and untargeted LC-MS/MS or UPLC-QTOF metabolomic analyses, polysaccharide structural characterization, and GC-MS analysis of volatile constituents. Activity-guided fractionation, followed by validation of individual compounds or defined chemical fractions, would help determine which aging-related constituents are linked to the observed biological effects. Second, the gut microbiota analysis was limited to 16S rRNA sequencing, including α-diversity, β-diversity, taxonomic relative abundance, and correlation analysis. This approach can describe microbial community shifts but cannot directly reveal microbial functional capacity, microbial metabolites, or causal host–microbe interactions. Although correlations were observed between selected genera and metabolic indicators, these associations do not prove that gut microbiota changes mediated the lipid-lowering effects of CRP extracts. Future studies should include shotgun metagenomic sequencing, fecal metabolomics or short-chain fatty acid analysis, and validation using antibiotic-treated or fecal microbiota transplantation models. Third, the HED calculated from the mouse dose was approximately 12.16 mg/kg body weight/day, equal to about 0.73–1.0 g/day of CRP extract for adults weighing 60–80 kg. Since the extraction yield of CRP extract in this study was approximately 30%, this estimated intake corresponds to about 2.4–3.3 g/day of dried CRP raw material. Such a gram-level intake appears achievable through standardized dietary supplements or functional foods enriched with CRP extract. Still, this calculation is only a translational estimate and should not be taken as a recommended human intake level. Human intervention studies are needed to verify the effective dose range, safety, and long-term compliance of aged CRP products. Fourth, the present study was conducted in mice, and human studies are needed to confirm the translational relevance of aged CRP as a functional food ingredient. In addition, RT-qPCR primer specificity was checked by melting curve analysis, whereas primer amplification efficiency was not determined by standard curve analysis. Future work should include standard curve-based efficiency validation to strengthen the quantitative reliability of RT-qPCR analysis. Finally, processing standardization, long-term safety, stability, and sensory characteristics should also be evaluated before commercial application.

In summary, improvements across the measured lipid metabolic parameters were more consistently observed in the longer-aged CRP extract groups than in the shorter-aged groups. This pattern supports enhanced metabolic functionality following postharvest aging. The improvement was accompanied by progressive decreases in hesperidin, nobiletin, and tangeretin, not by corresponding increases in these three markers. The stronger activity of longer-aged CRP extracts therefore appears to involve broader chemical changes within the CRP phytochemical matrix. Comprehensive metabolomic profiling and activity-guided validation will be needed to identify the active constituents and aging-related chemical transformations responsible for this effect.

## 5. Conclusions

Under the tested conditions, postharvest aging exerted a clearer influence on the metabolic functionality of CRP extracts than harvest stage. CRP-Q contained higher levels of hesperidin, nobiletin, and tangeretin than CRP-H at the same aging duration, whereas total sugar content was higher in CRP-H. These compositional differences, however, did not match the overall pattern of biological activity. Longer-aged CRP extracts, especially the three- and five-year-aged samples, produced more consistent improvements in HFD-induced lipid metabolic disturbances, including serum lipid abnormalities, hepatic steatosis, epididymal fat accumulation, adipocyte hypertrophy, lipid metabolism-related enzymes, and regulatory genes. Changes in SREBP-1c, HMGCR, PPAR-α, and PPAR-γ, together with altered FAS, ACC, HSL, and LPL levels, suggest that whole CRP extracts helped rebalance lipid synthesis and lipid catabolism.

The enhanced activity of longer-aged CRP extracts was not accompanied by increased hesperidin, nobiletin, or tangeretin. These three quantified flavonoids declined during aging, which indicates that they are insufficient to explain the stronger biological effects of aged CRP. Other uncharacterized constituents, such as minor flavonoids, phenolic acids, essential-oil or volatile constituents, terpenes, polysaccharide fractions, non-flavonoid compounds, and aging-derived transformation products, may contribute individually or through combined effects. Changes in selected gut microbial taxa, including Akkermansia, were observed together with improved metabolic indicators, but these findings remain associative. Further metabolomic profiling, polysaccharide and volatile-compound characterization, activity-guided validation, and deeper microbiota-related studies are needed before the chemical basis, causal mechanism, and functional-food potential of aged CRP can be fully defined.

## Figures and Tables

**Table 1:** Contents of hesperidin, nobiletin, tangeretin, and total sugar in CRP extracts from different harvest stages and aging durations.

Group	Hesperidin (mg/g)	Nobiletin (mg/g)	Tangeretin (mg/g)	Total Sugar (mg/g)
CRP-1Q	58.09 ± 0.91 ^a^	14.78 ± 0.18 ^a^	9.15 ± 0.08 ^a^	123.71 ± 8.93 ^a^
CRP-1H	55.60 ± 1.27 ^ab^	10.50 ± 0.06 ^bc^	6.65 ± 0.04 ^c^	281.08 ± 2.18 ^bc^
CRP-3Q	56.33 ± 0.81 ^ab^	13.42 ± 0.06 ^ab^	7.21 ± 0.03 ^b^	220.96 ± 2.59 ^ab^
CRP-3H	47.45 ± 0.45 ^bc^	10.19 ± 0.05 ^bc^	6.56 ± 0.17 ^c^	376.29 ± 7.78 ^de^
CRP-5Q	49.56 ± 0.28 ^abc^	11.51 ± 0.11 ^abc^	6.73 ± 0.04 ^c^	292.60 ± 2.78 ^bd^
CRP-5H	40.28 ± 0.65 ^c^	7.50 ± 0.04 ^c^	4.00 ± 0.03 ^d^	427.31 ± 14.71 ^e^

Values are expressed as mean ± SEM. CRP-1Q, CRP-3Q, and CRP-5Q represent green CRP extracts aged for 1, 3, and 5 years, respectively; CRP-1H, CRP-3H, and CRP-5H represent red CRP extracts aged for 1, 3, and 5 years, respectively. The sample sizes are indicated in the Section 2: Methods. Different superscript letters within the same column indicate significant differences among groups (*p* < 0.05).

## Data Availability

The data generated in this study are available from the corresponding author upon reasonable request.

## Supplementary Materials

The following supporting information can be downloaded at: https://www.mdpi.com/article/10.3390/foods15142470/s1. Table S1. Primer sequences used for RT-qPCR. Table S2. Effects of harvest stage, aging duration, and their interaction on chemical components, physiological parameters, lipid metabolism markers, gut microbiota diversity, and selected bacterial genera. Table S3. Semi-quantitative histopathological scoring of liver sections.

## Abbreviations

The following abbreviations are used in this manuscript:

ANOVA	ANOVA One-way analysis of variance
BW	Body Weight
CPT1A	Carnitine Palmitoyltransferase 1A
CRP	Citri Reticulatae Pericarpium
FA	Formic Acid
CRP-Q	Green Citri Reticulatae Pericarpium
GM	Gut Microbiota
H&E	Hematoxylin And Eosin
HMGCR	Hepatic 3-Hydroxy-3-Methylglutaryl-Coa Reductase
HDL-C	High-Density Lipoprotein Cholesterol
HFD	High-Fat Diet
HPLC	High-Performance Liquid Chromatography
LDL-C	Low-Density Lipoprotein Cholesterol
OTUs	Operational Taxonomic Units
PPAR-α	Peroxisome Proliferator-Activated Receptor α
RT-qPCR	Real-Time Quantitative Polymerase Chain Reaction
CRP-H	Red Citri Reticulatae Pericarpium
ROS	Reactive Oxygen Species
SREBP-1c	Sterol Regulatory Element-Binding Protein-1c
TC	Total Cholesterol
TCM	Traditional Chinese Medicine
TG	Triglycerides
