# Supplementary material for: Postharvest Aging and Harvest Stage Shape the Bioactive Profile, Hypolipidemic Activity, and Gut Microbiota-Modulating Effects of Citrus Peel Extracts in High-Fat-Diet-Fed Mice
Source: Foods. 2026 Jul 12;15(14):2470. doi: 10.3390/foods15142470 (PMC13409513; doi:10.3390/foods15142470)
Supplement: Supplementary file 1 [file foods-15-02470-s001.zip › foods-4383628-supplementary.pdf]

Supplementary Table S1. Primer sequences used for RT-qPCR.

Gene	Primer	Sequence（5'→3'）
SREBP-lc	Forward primer	GATCAAAGAGGAGCCAGTGC
	Reverse primer	TAGATGGTGGCTGCTGAGTG
HMGCR	Forward primer	TGACCTTTCTAGAGCGAGTGC
	Reverse primer	TGACATGGTGCCAACTCCAA
PPARα	Forward primer	TCTGGGCAAGAGAATCCACG
	Reverse primer	CAAAAGGCGGGTTGTTGCTG
PPARγ	Forward primer	GACCCAGCTCTACAACAGGC
	Reverse primer	CCCTTGCATCCTTCACAAGC
GAPDH	Forward primer	GGTTGTCTCCTGCGACTTCA
	Reverse primer	TGGTCCAGGGTTTCTTACTCC

Supplementary Table S2. Effects of harvest stage, aging duration, and their interaction on chemical components, physiological parameters, lipid metabolism markers, gut microbiota diversity, and selected bacterial genera.

Component	Harvest stage	Aging duration	Harvest × Aging
Hesperidin	****	****	***
Nobiletin	****	****	***
Tangeretin	****	****	****
Total sugar	****	****	ns
Liver weight	ns	ns	ns
Epididymal fat weight	ns	**	ns
Final body weight	ns	ns	ns
TC	ns	***	ns
TG	ns	ns	ns
LDL	ns	ns	ns
HDL	*	ns	ns
FAS	**	****	ns
ACC	*	***	ns
HSL	ns	****	ns
LPL	ns	****	ns
SREBP1c	**	**	*
HMGCR	*	**	**
PPARα	****	****	****
PPARγ	ns	ns	ns
Chao1	ns	*	ns
ACE	ns	*	ns
Shannon	ns	*	ns
Simpson	ns	ns	ns
Akkermansia	ns	*	ns
Lactobacillus	ns	ns	ns
Bacteroides	ns	**	ns

P values were obtained from two-way ANOVA with harvest stage and aging duration as fixed factors. Harvest × Aging indicates the interaction between harvest stage and aging duration. ns, $p >= 0.05$; *, $p < 0.05$; **, $p < 0.01$; ***, $p < 0.001$; ****, $p < 0.0001$

Supplementary Table S3. Semi-quantitative histopathological scoring of liver sections.

Group	Steatosis	Lobular inflammation	Ballooning	NAS total score
Control	0	0	0	0
HFD model	2	1	1	4
Positive control	1	0.33	1	2.33
CRP-1Q	1	0	0	1
CRP-1H	1	1	1	3
CRP-3Q	1	0	0	1
CRP-3H	0	1	0	1
CRP-5Q	1	1	0	2
CRP-5H	1	1	0.67	2.67

Note: Steatosis was scored from 0 to 3, lobular inflammation from 0 to 3, and hepatocellular ballooning from 0 to 2. The NAS total score was calculated as steatosis score + lobular inflammation score + hepatocellular ballooning score. Values represent the arithmetic means of three liver sections evaluated within each treatment group.
